# Surface Plasmon-Enhanced Photoelectrochemical Sensor Based on Au Modified TiO_2_ Nanotubes

**DOI:** 10.3390/nano12122058

**Published:** 2022-06-15

**Authors:** Wanqing Liu, Wei Duan, Liqun Jia, Siyu Wang, Yuan Guo, Guoqing Zhang, Baolin Zhu, Weiping Huang, Shoumin Zhang

**Affiliations:** 1 Department of Chemistry, Key Laboratory of Advanced Energy Material Chemistry (MOE), Tianjin Key Laboratory of Metal and Molecule Based Material Chemistry, Nankai University, Tianjin 300071, China; 17803870886@163.com (W.L.); dduan0417@163.com (W.D.); jlq15064153315@163.com (L.J.); siyu2142021@163.com (S.W.); 2120190685@mail.nankai.edu.cn (Y.G.); zhangguoqing9401@163.com (G.Z.); hwp914@nankai.edu.cn (W.H.); 2 National Demonstration Center for Experimental Chemistry Education, Nankai University, Tianjin 300071, China

**Keywords:** Au nanoparticles, TiO_2_ nanotubes, SPR effect, glucose PEC sensor, visible red light

## Abstract

Based on the enhanced charge separation efficiency of the one-dimensional structure and strong surface plasmon resonance (SPR) of gold, a gold modified TiO_2_ nanotube (Au/TiO_2_NTs) glucose photoelectrochemical (PEC) sensor was prepared. It could be activated by visible red light (625 nm). Under optimal conditions, the Au/TiO_2_NTs sensor exhibited a good sensitivity of 170.37 μA·mM^−1^·cm^−2^ in the range of 1–90 μM (R^2^ = 0.9993), and a detection limit of 1.3 μM (S/N = 3). Due to its high selectivity, good anti-interference ability, and long-term stability, the fabricated Au/TiO_2_NTs sensor provides practical detection of glucose. It is expected to be used in the construction of non-invasive PEC biosensors.

## 1. Introduction

Glucose is one of the body’s primary energy sources and an important biomarker in clinical diabetes diagnosis. Diabetes is a severe disease that can lead to severe complications, such as blindness, lower limb amputations, and cardiovascular disease. Sometimes, it can even lead to death. According to the latest report, the number of adults with diabetes worldwide reached 537 million in 2021, with one person dying from diabetes every five seconds on average. Although there is no effective cure for diabetes, complications associated with the disease can be reduced by tightly controlling blood sugar levels. Therefore, it is significant to explore fast and reliable glucose concentration monitoring methods in the treatment of diabetes [1]. Among the reported methods for detecting glucose, photoelectrochemical (PEC) technology has been widely considered for its simple operation, high sensitivity, low detection limit, high specificity, low cost, and so on [2,3,4]. The PEC process is generally composed of photoelectric conversion and an electrochemical process [5]. The analyte changes the properties of the photosensitive material or the electrolyte environment, resulting in a change in the PEC signal intensity; thereby realizing quantitative analysis of the analyte concentration [6].

As the central part of PEC biosensors, the semiconductor materials play the role of molecular fixation, signal generation, and transmission in the system [7,8]. Therefore, the photoelectric properties of the used semiconductors are vital for the performance of PEC sensors. Many photoactive semiconductors with excellent PEC performance have been investigated and applied [9,10].

TiO_2_ is considered to be the optimal and typical semiconductor nanomaterial for the construction of PEC biosensors, due to its great photocatalytic performance, good stability, non-toxicity, adjustable morphology, and good biocompatibility [11,12]. It is widely used in photocatalysis [13], solar cells [14], and PEC sensors [15], etc. The nano-morphology of TiO_2_ includes nanosheet [16], nanocrystal [17], nanoarray [18], nanoparticle [19], nanorod [20], nanowire [21], nanoneedle [22,23], nanoflower [24], nanocube [25], and monocrystal forms [26,27]. Due to the difference in morphology, the materials show different physical and chemical properties, and their applications in bioanalytical sensing are also commonly reported [28]. Among all the structures of TiO_2_, many reports have indicated that a one-dimensional (1D) TiO_2_ nanotube structure can accelerate the separation efficiency of e^−^ and h^+^ pairs, due to the transport of electrons along the nanotube axis. Thus, electrons are collected efficiently at the junction, and e^−^ and h^+^ pairs are not easily combined [29,30]. What is more, compared with other 1D nanostructures, TiO_2_ nanotubes have a larger specific surface area; that is, they can provide more active centers, which gives TiO_2_ nanotubes better PEC performance.

However, TiO_2_ nanotubes also have their defects. The bandgap determines the wavelength of the excitation light source. Pure TiO_2_ can only be excited by light in the ultraviolet region (UV; λ < 387 nm), due to its wide bandgap (3.2 eV) [31]. It is worth noting that ultraviolet light is harmful to most biomolecules [32]. The low penetration depth of shortwave excitation sources to biological tissues also limits the application of PEC biosensors in living organism analysis. In addition, the difficulty of surface charge transfer and the easy recombination of electron-hole pairs are also factors that limit the ultimate sensitivity of PEC sensing. Through different modification methods, the PEC performance of TiO_2_ can be effectively improved. Based on the unique photoelectronic properties of TiO_2_, advanced photoelectronic materials with high-performance PEC biosensors have been developed, by forming heterojunctions [33], introducing wide bandgap semiconductors [34], loading noble metals, and defect engineering methods [35].

In recent years, it has been found that noble metal nanoparticles with surface plasmon resonance (SPR) characteristics can improve the optical conversion efficiency of TiO_2_ [36]. In particular, gold nanoparticles (AuNPs), because of their good stability, biocompatibility, synthetic controllability, and excellent catalytic performance [37], have become the research hotspot of SPR-enhanced optical activity [38,39]. When gold nanoparticles interact with the incident light field, the collective excitation of conduction electrons will lead to local surface plasmon resonance (LSPR). Liu et al. introduced a novel plasmonic device that combined the concepts of a perfect absorber and an LSPR sensor, which remained highly absorptive over a wide range of incident angles for both transverse electric (TE) and transverse magnetic (TM) configurations [40]. Zaman and his team mapped and analyzed the optical forces generated by a right-handed plasmonic Archimedean spiral (PAS), which could be used in the initial design process of complex laboratory-on-a-chip systems [41]. Wu et al. reported that highly dispersed AuNPs were modified on the surface of TiO_2_ nanotube arrays by pulse electrodeposition technology, which could produce the SPR effect and increase the absorption of visible light, to improve the PEC performance of TiO_2_ [42]. Melvin and his team reported that Au-Pt alloy was supported on the surface of TiO_2_, and the bimetal was used as an electron absorbent to weaken the rapid recombination of the carrier [43]. In addition, due to the strong SPR effect of noble metals, modification of AuNPs can broaden TiO_2_ light response to the visible red light range.

Visible red light has many advantages over ultraviolet and other visible light, due to its long wavelength. Biological samples primarily absorb or scatter short-wavelength light, so long-wavelength excitation can eliminate the interference from biological samples. Ali et al. reported that the transmittance of visible red light through human tissues was about 30 times higher than that of near-infrared light. In addition, the refractive index of the visible red light has a higher sensitivity to changes in glucose concentration, achieving higher linearity, faster response time, and accuracy [44].

In this work, AuNPs were modified on TiO_2_NTs, to fabricate a sensitive sensor of glucose, which could be excited by visible red light at 625 nm. The introduction of gold improved the optical conversion efficiency of TiO_2_ and expanded its light absorption range. The Au/TiO_2_NTs sensor exhibited high sensitivity, excellent linearity, low detection limit, good selectivity, and stability, and it is expected to be used for non-invasive blood glucose detection.

## 2. Materials and Methods

### 2.1. Materials

Titanium dioxide power (TiO_2_) was purchased from Shanghai Aladdin Industrial Corporation, (Shanghai, China). Tetrabutyl titanate (C_16_H_36_O_4_Ti), glucose (C_6_H_12_O_6_∙H_2_O), and sodium hydroxide (NaOH) were purchased from Tian Jin Jing Dong Tian Zheng, (Tianjin, China). Nitric acid (HNO_3_) and absolute ethanol were procured from Tianjin Guangfu, (Tianjin, China). Nafion solution (20 wt.%) was procured from Tianjin Yifang Technology Co. Ltd., (Tianjin, China). Chlorauric acid tetrahydrate (HAuCl_4_·4H_2_O) was bought from Tianjin Guangfu Fine Chemical Research Institute, (Tianjin, China). Saccharose and fructose (99%) were purchased from Shengfei Biotechnology Service Center, Nankai District, Tianjin, China. Lactose (AR), uric acid (99%), and ascorbic acid (AR) were procured from Tianjin Jiangtian Chemical Technology Co., Ltd., (Tianjin, China). All chemicals in this study were analytical grade and could be used without further purification. All experiments were conducted with ultrapure water.

### 2.2. Preparation of Titanium Dioxide Nanotubes (TiO_2_NTs)

The hydrogen titanate nanotubes (HTNTs) and stabilized TiO_2_NTs were prepared according to our previous work [45]. Briefly, pure anatase TiO_2_ powder was treated by the hydrothermal method in 10 M NaOH solution at 150 °C for 12 h, and then washed with ultrapure water to pH = 7. Then, 0.1 mol/L HNO_3_ was added to the solution until pH = 2~3. Then, the obtained HTNTs were washed with ultrapure water until the solution was neutral. After centrifugation, the obtained sample was dried at 80 °C for 10 h.

Stabilized TiO_2_NTs were synthesized using the sol impregnation method, with HTNTs as the precursors. HTNTs were added in titanium sol and stirred for 4 h. After drying at room temperature, the nanotubes were calcined at 400 °C in the air for 2 h to obtain TiO_2_NTs.

### 2.3. Preparation of Au/TiO_2_NTs

The gold-modified TiO_2_NTs were prepared by a photoreduction process. First, 0.3 g TiO_2_NTs were dispersed in a mixed solution of 30 mL anhydrous ethanol and 30 mL deionized water, stirred for 0.5 h, and ultrasonically rinsed for 0.5 h. The above suspension was then bubbled with nitrogen for 15 min, to remove the dissolved oxygen. Then, different volumes of 0.01 mol/L HAuCl_4_·4H_2_O solution were added, and the mass percentage of gold was 0.25, 0.50, 0.75, and 1.0 wt.%, respectively. Subsequently, the suspension was irradiated under ultraviolet light (300 W Hg lamp) for 2.5 h. Finally, the prepared samples were centrifugated and washed several times, and then dried at 80 °C for 8 h. According to the mass percentage of Au, the obtained samples were marked as 0.25, 0.50, 0.75, and 1.0 wt.% Au/TiO_2_NTs, respectively. The actual gold loadings in Au/TiO_2_NTs were 0.193, 0.356, 0.519, and 0.746 wt.%, respectively. This can be attributed to the gold losses in the preparation process.

### 2.4. Fabrication of Au/TiO_2_NTs Photoelectrode

The 4-mg as-prepared samples were first dissolved in the mix solution of 720 μL deionized water, 240 μL anhydrous ethanol, and 40 μL 5% Nafion solution. Then the content was ultrasonicated for 0.5 h, to obtain a homogenous suspension. Then, 10 μL of suspension was dropped on the surface of a glassy carbon electrode (GCE, 4 mm in diameter) and dried at room temperature. The coated GCE was denoted as Au/TiO_2_NTs photoelectrodes.

### 2.5. Characterization

X-ray diffraction (XRD) was characterized using a diffractometer (Rigaku SmartLab, Rigaku Corporation, Tokyo, Japan). Transmission electron microscope (TEM) images were observed by a Talos F200X G2 instrument. X-ray Photoelectron Spectroscopy (Kratos Axis Ultra DLD, Kratos Analytical Ltd., Manchester, UK) recorded the chemical composition and oxidation state of the elements of samples. The actual Au loadings on the catalysts were examined using an inductive coupled plasma emission spectrometer (ICP-OES, Spectroblue, Spectro, Kleve, Germany). The UV–Vis diffuse reflection spectra (UV–Vis DRS) were recorded on a UV–Vis spectrophotometer (UV3600-Plus, Shimadzu, Kyoto, Japan). Photoluminescence spectra (PL) were recorded on a spectrophotometer (PTI, New York, NY, USA). Electrochemical measurements were performed on a Zahner Zennium electrochemical workstation (Kronach–Gundelsdorf, Germany). An XPA-7 photocatalytic device was from XuJiang Electromechanical Plant, Nanjing, China, which was used in the photoreduction process.

### 2.6. Photoelectrochemical Measurements

The photoelectrochemical measurements were performed on a workstation via a three-electrode system at room temperature. The platinum wire electrode and Ag/AgCl electrode were used as counter electrode and reference electrode, respectively, while Au/TiO_2_NTs nanocomposite modified GCE (4 mm diameter) was utilized as a working electrode. The electrolyte was composed of 0.1 M NaOH solution in a 50-mL electrolytic cell. A LED lamp (625 nm, 30.98 W/m^2^) was selected as the illumination source during the PEC testing process.

## 3. Results and Discussion

### 3.1. Structure Characterization by XRD

**Figure 1:**
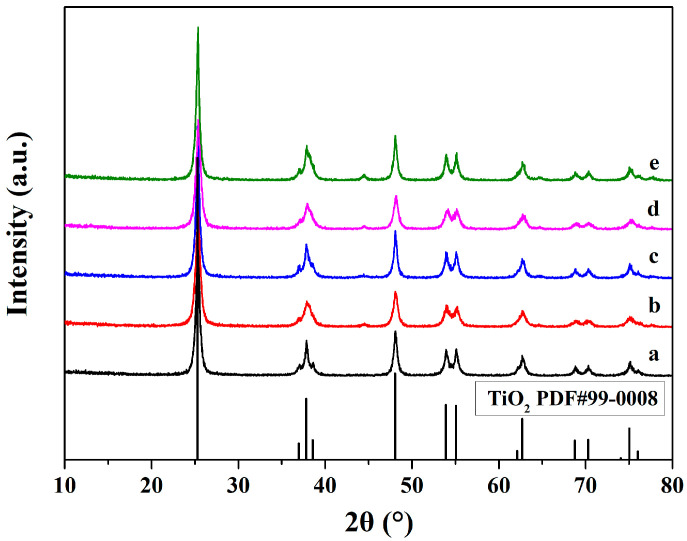
XRD pattern of (**a**) TiO_2_NTs and Au/TiO_2_NTs with different gold contents: (**b**) 0.25 wt.%, (**c**) 0.5 wt.%, (**d**) 0.75 wt.%, and (**e**) 1.0 wt.%.

The XRD measurement was carried out to explore the materials’ phase. Figure 1 shows the XRD patterns of the pure TiO_2_NTs and the Au/TiO_2_NTs with different gold contents (0.25, 0.5, 0.75, and 1.0 wt.%). As shown in Figure 1, the as-prepared TiO_2_NTs were anatase phase, with principal diffraction peaks at 2θ = 25.3°, 36.9°, 37.8°, 38.6°, 48.0°, 53.9°, 55.1°, 62.1°, 62.7°, 68.8°, 70.3°, 74.0°, and 75.0°, which corresponded to the (101), (103), (004), (112), (200), (105), (211), (213), (204), (116), (220), (107), and (215) diffraction planes, respectively (JCPDS NO.99-0008). At the same time, only the diffraction peaks of pure TiO_2_ were observed, and no diffraction peak related to metal Au was observed, which could be attributed to the relatively low loading of Au and the high dispersion in TiO_2_NTs. In addition, the diffraction peaks of the photocatalyst were sharp and high, indicating that the samples had good crystallinity. There were no diffraction peaks of other phases in Figure 1, indicating that the synthesized photocatalysts had good purity.

### 3.2. Morphology Characterization of Samples by TEM

**Figure 2:**
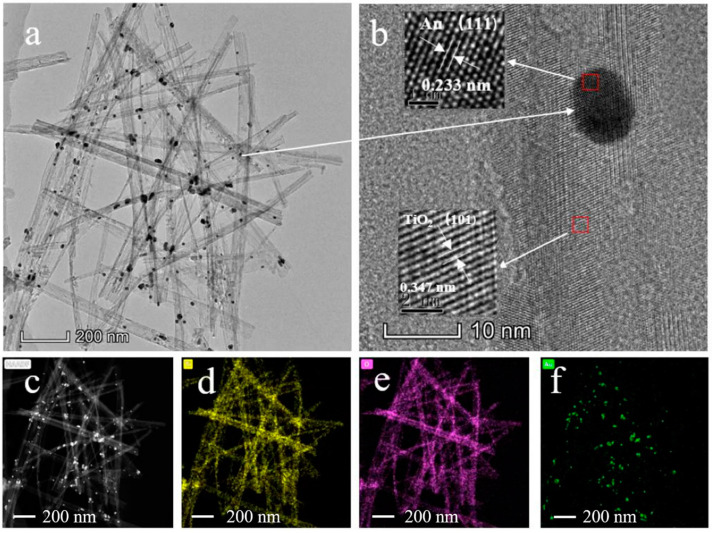
(**a**) TEM, (**b**) HRTEM, and (**c**) EDS mapping, (**d**) Ti, (**e**) O, (**f**) Au images of 0.5 wt.% Au/TiO_2_NTs.

The microstructure of the as-prepared Au/TiO_2_NTs was characterized by TEM. From the TEM images, nearly uniform nanotube-like structures could be clearly seen. The Au/TiO_2_NTs were hundreds of nanometers long, and the outer diameter was about 10 nanometers. As seen from Figure 2a, the black dots were confirmed to be AuNPs, with a size of about 8–9 nm. Figure 2b was the high-resolution HRTEM image of Au/TiO_2_NTs, in which the lattice fringes of 0.347 nm and 0.233 nm correspond well to anatase TiO_2_ (101) and Au (111) interfaces, respectively. It could be observed that the Au modification did not influence the nanotubes’ structure. Furthermore, EDS element mapping diagrams of Au/TiO_2_NTs were performed (Figure 2c–f). Except for the large gold particles, large amounts of gold particles with very small sizes can also be observed. They were highly dispersed on the nanotubes. As a result, Au/TiO_2_NTs with sufficient gold loading were obtained.

### 3.3. The Chemical State and Composition Analysis Using XPS

**Figure 3:**
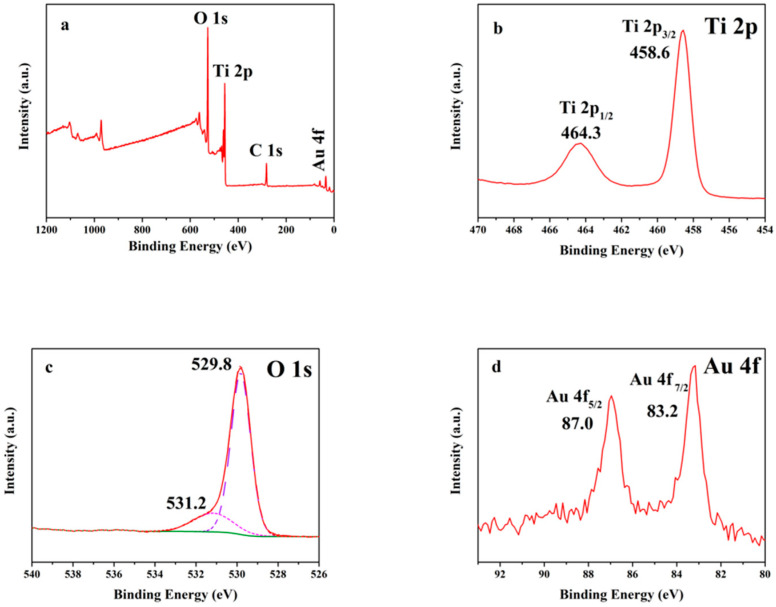
(**a**) XPS full spectrum of 0.5 wt.% Au/TiO_2_NTs, and high-resolution spectrum for (**b**) O 1s; (**c**) Ti 2p; (**d**) Au 4f.

To further study the electronic interaction between TiO_2_ and Au, XPS was performed. The full XPS spectrum is shown in Figure 3a, in which the electronic states of Ti 2p, O 1s, C 1s, and Au 4f can be observed. The mass percentage of Au in the 0.5 wt.% Au/TiO_2_NTs was 0.369 wt.%, which is consistent with the result obtained by ICP. The C signal might come from the XPS measurement itself. Figure 3b shows the Ti XPS spectrum. The binding energy of Ti 2p_1/2_ and Ti 2p_3/2_ were located at 464.3 eV and 458.6 eV, respectively, indicating that Ti exists in the +4 valence state [46,47]. It corresponded to the existence of stoichiometric TiO_2_. The O 1s XPS spectrum showed two peaks at a binding energy at 531.2 eV and 529.8 eV (Figure 3c), respectively, and belonging to Ti-OH and lattice oxygen [Ti-O_6_] species, respectively [48,49]. Figure 3d shows the Au 4f XPS spectrum of Au/TiO_2_NTs. It can be clearly observed that the samples showed a main Au 4f_7/2_ peak at 83.2 eV, with an analogous Au 4f_5/2_ peak at 87.0 eV, which indicated that gold in the Au/TiO_2_NTs was presented in the metallic state [50]. In addition, the peak of Au 4f was negatively shifted compared with the characteristic peak of standard Au (83.7 eV), indicating that Au was electron-rich, which may have been caused by e^−^ transfer from the carrier to gold. This showed that there was a strong interaction between TiO_2_ and gold. Peaks of oxidized gold species were not observed, which should be around 86.3 and 85.5 eV. It seemed that the trivalent gold in chloroauric acid had been reduced to elemental gold after the photoreduction process.

### 3.4. Optical Absorption Properties Analysis by UV–Vis DRS

**Figure 4:**
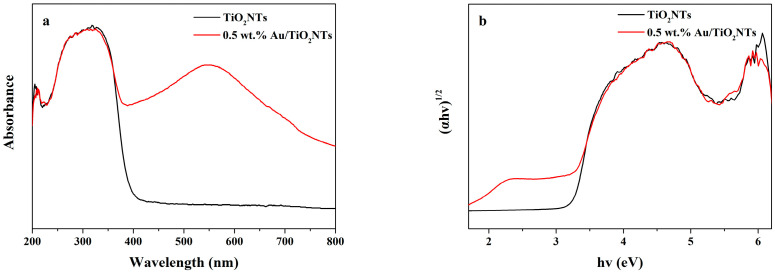
(**a**) UV–Vis DRS, and (**b**) bandgap energies of TiO_2_NTs and 0.5 wt.% Au/TiO_2_NTs.

To further analyze the optical absorption ability of the material, the UV-Vis DRS test was selected to characterize the samples. It could be clearly seen from the UV-Vis DRS spectrum that both TiO_2_NTs and Au/TiO_2_NTs had a strong absorption peak at 230–380 nm. It was confirmed that the loading of noble metals did not change the inherent absorption of TiO_2_. Combined with Figure 4a,b, it could be seen that the absorption edge of TiO_2_ nanotubes had a redshift after being loaded with gold. The corresponding bandgap widths of TiO_2_NTs and Au/TiO_2_NTs were 3.2 and 3.0 eV, respectively. As the absorption edge expanded, the bandgap decreased; that is, the spectrum range that TiO_2_NTs could respond to after loading the noble metal became significantly wider. This could improve the deficiency of TiO_2_ catalyst and help the photocatalyst to capture more wavelengths of light; thus, generating more photogenerated electrons to participate in a photoelectric chemical reaction. Moreover, obvious absorption peaks in the visible light range of 500–600 nm could be observed in Au/TiO_2_NTs catalysts, which were attributed to the SPR effect of the loaded AuNPs [51]. As a result, the loaded AuNPs could significantly improve the absorption of TiO_2_NTs under visible light and improve the PEC performance of TiO_2_NTs.

### 3.5. The Optical Properties Analysis by Photoluminescence (PL) Spectroscopy

**Figure 5:**
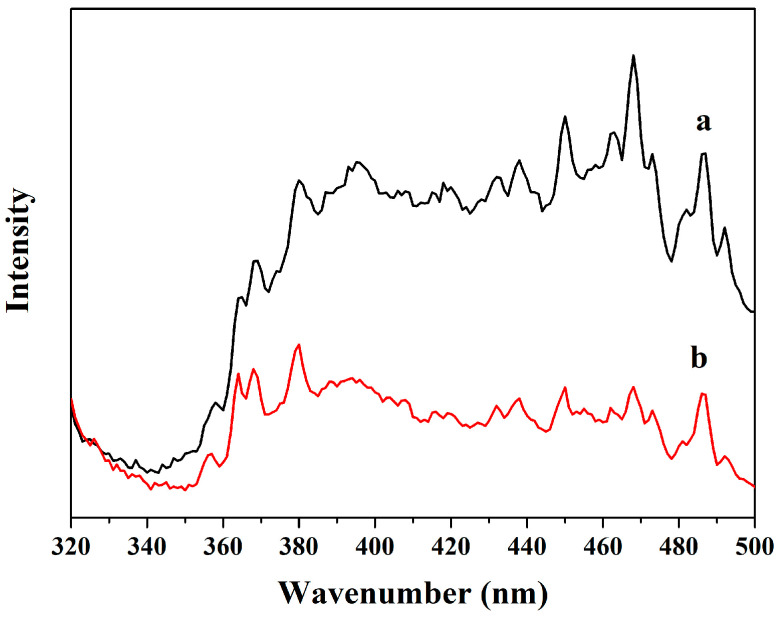
Photoluminescence spectra of (**a**) TiO_2_NTs and (**b**) 0.5 wt.% Au/TiO_2_NTs in the powder form at 300 nm excitation.

The recombination ability of e^−^ and h^+^ was characterized by photoluminescence intensity. As displayed in Figure 5, the photoluminescence spectra of the samples were evaluated at the excitation wavelength of 300 nm. The PL intensity of Au/TiO_2_NTs was lower than that of pure TiO_2_NTs, which indicated that the loading of gold was beneficial for inhibiting the recombination of e^−^ and h^+^ and improving the photoelectric performance. As indicated in the XPS spectra, the formation of the Schottky junction enabled the gold to become electron-rich. Thus, the e^−^ and h^+^ pairs could be effectively separated, and the charge recombination could be avoided. Therefore, the 0.5 wt.% Au/TiO_2_NTs have a higher photogenerated carrier separation rate and mobility than TiO_2_NTs, which was beneficial for generating more photogenerated e^−^ and h^+^, and exhibiting better PEC performance than the pure TiO_2_NTs.

### 3.6. Photoelectrochemical Measurements

#### 3.6.1. The Influence of Potential and Au Load on the Performance of Biosensing

**Figure 6:**
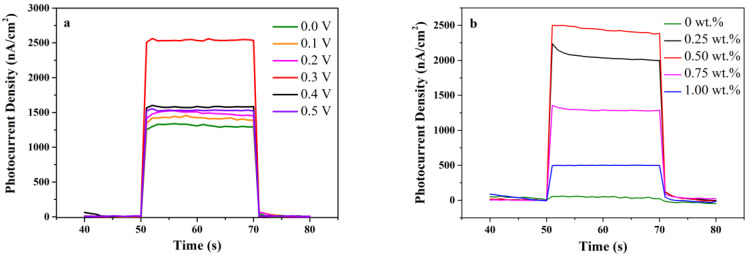
(**a**) Photocurrent–time responses at different bias voltages in 0.1 M NaOH, and (**b**) photocurrent–time responses of different gold loading of Au/TiO_2_NTs in 0.1 M NaOH at 0.3 V.

A photocurrent–time curve can characterize the separation efficiency of the photogenerated carriers. Experimental parameters may affect PEC performance. Thus, the experimental parameters, such as bias voltages and gold contents, must be optimized. The application of bias voltages on the photoelectrode can facilitate the separation of e^−^ and h^+^ and reduce their recombination rate, which can be directly reflected in the enhancement of photocurrent. The influence of voltages on the photocurrent of Au/TiO_2_NTs electrode under the irradiation of 0.1 M NaOH solution at 625 nm was investigated. In the range of 0–0.5 V, the maximum photocurrent response was at 0.3 V (Figure 6a). Therefore, 0.3 V was determined as the optimum bias voltage. In addition, it is necessary to optimize the effect of gold content on photocurrent response. Under the irradiation of 625 nm, the influence of gold loading content was studied in 0.1 M NaOH solution. It can be seen from Figure 6b that no significant photocurrent was observed on the bare TiO_2_NTs, while an obvious photocurrent could be observed on the gold-modified ones. Due to its wide bandgap, TiO_2_ only could absorb short-wavelength ultraviolet light. As the gold contents increased, the photocurrent intensity first increased, reaching a maximum value of 0.5 wt.% Au/TiO_2_ NTs, and then gradually decreased.

In the Au/TiO_2_NTs, as a result of the high work function of gold, the electrons migrate from TiO_2_ to gold. When the Fermi levels of the gold and TiO_2_ are equal, a Schottky barrier will form. Consequently, the electrons and holes generated after illumination can be localized on gold and TiO_2_, respectively, be separated, and undergo oxidation-reduction reactions at different positions. When glucose exists in the system, the holes on the electrode surface will react with the glucose in the solution, and glucose is easily oxidized. When the gold content is too low, there are not enough electron receiving centers for effective e^−^ and h^+^ separation. However, when the gold content is too high, the gold coating on the surface of TiO_2_ has a shielding effect and affects the acceptance of electrons by TiO_2_. Moreover, the further deposition of gold results in the growth of AuNPs, which leads to the aggregation of gold atoms, the reduction of active sites, and the formation of recombination centers for e^−^ and h^+^ on gold particles. In addition, with too much gold loading, the color of the sample is too dark and this leads to light avoidance, resulting in a decrease in the photocurrent. Therefore, Au/TiO_2_NTs with 0.5 wt.% gold content exhibited the best PEC performance in this system.

#### 3.6.2. PEC Biosensing Application for Glucose

**Figure 7:**
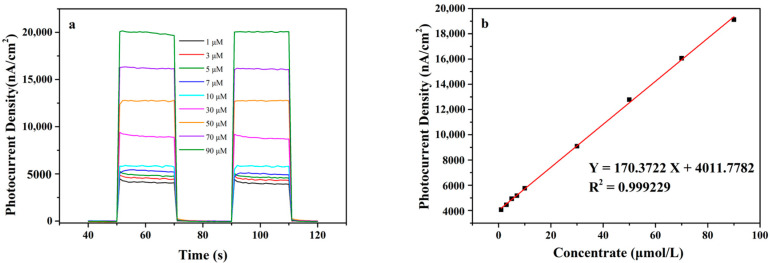
(**a**) Photocurrent curve in different concentrations of glucose, and (**b**) a corresponding calibration curve of the 0.5 wt.% Au/TiO_2_NTs electrode for the detection of glucose (1–90 μM).

Figure 7a shows the photocurrent curve on the Au/TiO_2_NTs electrode in different concentrations of glucose (1–90 μM). It can be observed that the intensity of photocurrent increased with the increase of the concentrations of glucose. In essence, they exhibited a linear regression equation: j (nA/cm^2^) = 4011.7782 + 170.3722 Cglucose (μM) with a correlation coefficient of 0.9993, where j is photocurrent density, and C is the concentration of the glucose (Figure 7b). The corresponding sensitivity of the Au/TiO_2_NTs biosensor was 170.37 μA·mM^−1^·cm^−2^. The detection limit (LOD) of the biosensor was 1.3 μM, which was obtained from LOD = 3sb/S, (sb represents standard deviation of blank signal, and S represents sensitivity).

Table 1 summarizes the comparison of different glucose biosensors. Compared with the reported PEC sensors, the Au/TiO_2_NTs in this work exhibited a long excitation wavelength, high sensitivity, and low LOD. Most excitation light sources in PEC sensor research were ultraviolet or visible light with short wavelengths, which could cause cell damage or degeneration. In our work, visible red light with a wavelength of 625 nm was selected as the excitation wavelength. This low-energy long-wavelength light is more conducive to noninvasive blood glucose monitoring than short-wavelength light.

At present, blood analysis is the most commonly used method to monitor blood glucose, which has the characteristics of invasiveness, infection, bleeding, complex operation, and low efficiency. Compared to the traditional blood glucose technique, the high sensitivity and low LOD of the Au/TiO_2_NTs PEC sensors are beneficial to the realization of in vitro detection and provide the possibility of non-invasive glucose detection for humans, such as using interstitial fluid and sweat.

#### 3.6.3. PEC Biosensing Selectivity and Stability Analysis

**Figure 8:**
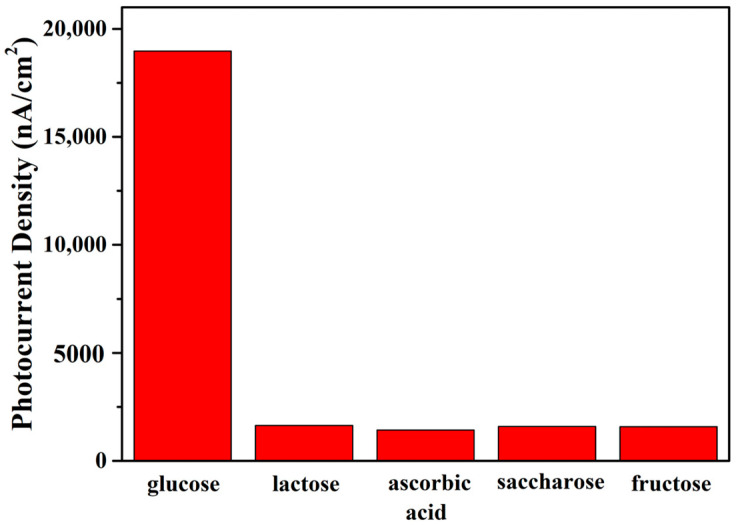
Comparison of photocurrent sizes of a 0.5 wt.% Au/TiO_2_NT electrode in various solutions.

Selectivity is an important parameter of biosensors. Figure 8 shows the photocurrent comparison of a 0.5 wt.% Au/TiO_2_NTs electrode in glucose, sucrose, lactose, ascorbic acid, saccharose, and fructose solutions (the concentration of the solution was 90 μM). It was obvious that the photocurrent generated on the Au/TiO_2_NTs electrode in the glucose solution was much greater than in other solutions, indicating that the Au/TiO_2_NT PEC sensor had a good selectivity.

**Figure 9:**
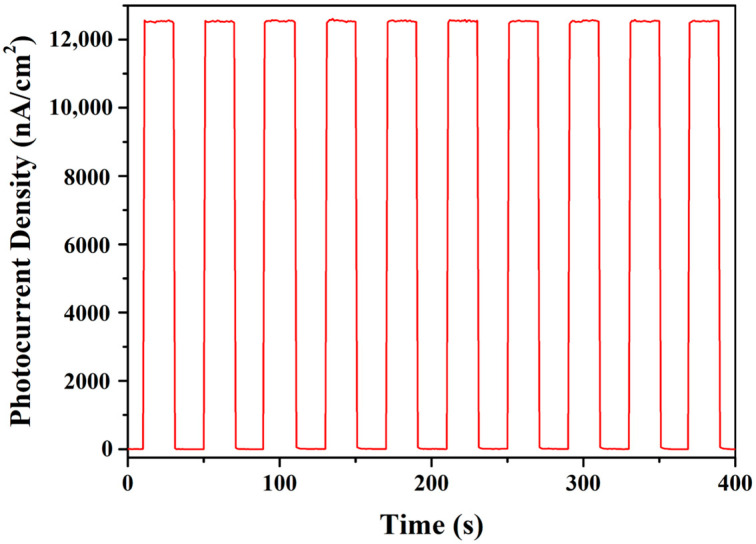
Photocurrent response stability of the 0.5 wt.% Au/TiO_2_NT electrode in 50 μM glucose solution.

Furthermore, the photocurrent response stability of the Au/TiO_2_NT electrode was investigated by switching the i–t response 10 times. As can be seen from Figure 9, the photocurrent increased rapidly at the moment of turning on the light and became stable within a few seconds. With switching 10 times, the photocurrent remained stable, and the photocurrent intensity had no obvious change in each cycle. The results showed that the Au/TiO_2_NTs had a good photocurrent response stability.

**Figure 10:**
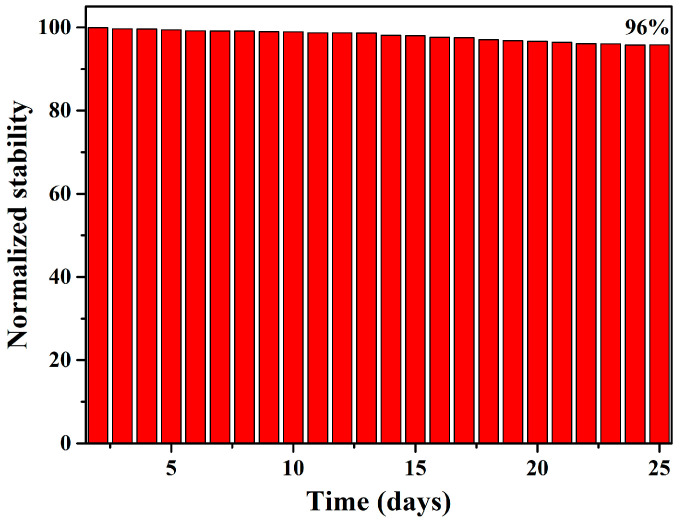
Relationship between the times and response of the 0.5 wt.% Au/TiO_2_NTs in 50 μM glucose solution.

Long-term stability is another important parameter of sensor performance. The 0.5 wt.% Au/TiO_2_NT electrode was stored in a room temperature atmosphere, and the photocurrent–time curve was continuously tested every day in a 50 μM glucose solution. Figure 10 shows the data for 25 days. It can be observed that the photocurrent intensity of the Au/TiO_2_NTs electrode maintained 96% of its original photocurrent intensity, indicating that the Au/TiO_2_NTs glucose sensor had a good long-term stability.

**Scheme 1:**
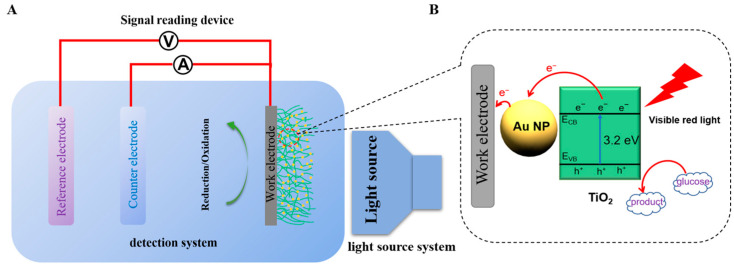
(**A**) Photoelectrochemical measurements via the three-electrode system; (**B**) the mechanism for PEC detection of glucose on the Au/TiO_2_NTs.

To clarify the PEC process of Au/TiO_2_NTs, we elucidate the mechanism of action of the glucose sensor in Scheme 1. The modification of AuNPs plays a key role in the PEC-generated e^−^ and h^+^ in TiO_2_. The SPR effect of AuNPs enhances the light absorption of TiO_2_ under visible light. In addition, the large gold work function causes the Au/TiO_2_NTs interface to form a Schottky barrier. Therefore, the photoinduced electrons are easily transferred to the gold particles, thereby decreasing the recombination of e^-^ and h^+^ and accelerating the separation efficiency of photoinduced carriers. Assisted by a bias voltage between the counter electrode and the working electrode, electrons are transferred along AuNPs to the glassy carbon electrode and eventually to the counter electrode via an external circuit. The holes on the Au/TiO_2_NTs electrode directly oxidize the glucose molecules. During this process, the glucose concentration changes, resulting in a change in the photocurrent. Consequently, the linear relation between photocurrent and glucose concentration is presented.

## 4. Conclusions

To sum up, a high-sensitivity and SPR-enhanced PEC sensor based on TiO_2_NTs was fabricated, in which Au NPs were decorated on TiO_2_NTs using the photoreduction method. Via gold modification, the absorption of TiO_2_ under visible red light was promoted, attributable to the SPR effect, and the separation of e^−^ and h^+^ was promoted through the formation of the Schottky junction. The resultantly enhanced electrochemical performance produced the high sensitivity of the Au/TiO_2_NTs for glucose under visible red light. In addition, the low detection limit, high selectivity, and long-term stability for glucose provide the possibility of the noninvasive detection of glucose. In addition to glucose, we predict that this PEC sensor has potential applications for other substances, such as glutathione and dopamine.

## Figures and Tables

**Table 1:** Comparison of analytical characteristics of different glucose sensors.

Configuration of Biosensor	Linear Range (μM)	Sensitivity (μA·mM^−1^·cm^−2^)	Detection Limit (μM)	Ref.
Au/TiO_2_ NTs/GCE	1–90	170.37	1.3	This work
GODx/Au@C/TiO_2_/FTO	100–1600	29.76	49	[52]
GOx@Au-PANI-TiO_2_	2000–36000	0.83	20	[53]
ZnO NRs/Au NPs	10–2000	22	10	[54]
Cu_2_O/TiO_2_	3000–9000	14.56	62	[55]
GOx/TiO_2_	500–3000	9.25	15	[56]
GOx–Chitosan/TiO_2_NTAs	300–1500	5.46	70	[57]

## Data Availability

The data is available on reasonable request from the corresponding author.

## References

[1] Li W.B., Jiang D.S., Yan P.C., Dong J.T., Qian J.C., Chen J.P., Xu L. (2019). Graphitic carbon nitride/α-Fe_2_O_3_ heterostructures for sensitive photoelectrochemical non-enzymatic glucose sensor. Inorg. Chem. Commun..

[2] Çakıroğlu B., Özacar M. (2020). A photoelectrochemical biosensor fabricated using hierarchically structured gold nanoparticle and MoS_2_ on tannic acid templated mesoporous TiO_2_. Electroanalysis.

[3] He L.H., Liu Q.B., Zhang S.J., Zhang X.T., Gong C.L., Shu H.H., Wang G.J., Liu H., Wen S., Zhang B.Q. (2018). High sensitivity of TiO_2_ nanorod array electrode for photoelectrochemical glucose sensor and its photo fuel cell application. Electrochem. Commun..

[4] Zhang Y., Wang Q., Liu D., Wang Q., Li T., Wang Z. (2020). Cu_2_O-BiOI isotype (pp) heterojunction: Boosted visible-light-driven photoelectrochemical activity for non-enzymatic H_2_O_2_ sensing. Appl. Surf. Sci..

[5] Zhang K., Lv S., Zhou Q., Tang D. (2020). CoOOH nanosheets coated g-C_3_N_4_/CuInS_2_ nanohybrids for photoelectrochemical biosensor of carcinoembryonic antigen coupling hybridization chain reaction with etching reaction. Sens. Actuators B.

[6] Sivula K., Van De Krol R. (2016). Semiconducting materials for photoelectrochemical energy conversion. Nat. Rev. Mater..

[7] Yan P.C., Jiang D.S., Tian Y.H., Xu L., Qian J.C., Li H.N., Xia J.X., Li H.M. (2018). A sensitive signal-on photoelectrochemical sensor for tetracycline determination using visible-light-driven flower-like CN/BiOBr composites. Biosens. Bioelectron..

[8] Zhang S.P., Xu G.F., Gong L.S., Dai H., Li Y.L., Hong Z.S., Lin Y.Y. (2015). TiO_2_-B nanorod based competitive-like non-enzymatic photoelectrochemical sensing platform for noninvasive glucose detection. J. Mater. Chem. B.

[9] Shu J., Tang D.P. (2017). Current advances in quantum-dots-based photoelectrochemical immunoassays. Chem.—Asian J..

[10] Liras M., Barawi M., Víctor A. (2019). Hybrid materials based on conjugated polymers and inorganic semiconductors as photocatalysts: From environmental to energy applications. Chem. Soc. Rev..

[11] Zhang J.J., Zhou Y.Z., Zheng G.P., Huang Q.Y., Zheng X.C., Liu P., Zhang J.M., Guan X.X. (2016). Novel assembly and electrochemical properties of anatase TiO_2_-graphene aerogel 3D hybrids as lithium-ion battery anodes. Chem. Phys. Lett..

[12] Zang Y., Fan J., Ju Y., Xue H.G., Pang H. (2018). Current advances in semiconductor nanomaterial-based photoelectrochemical biosensing. Chem.-A Eur. J..

[13] Li J., Li M., Gui P., Zheng L.N., Liang J.S., Xue G. (2019). Hydrothermal synthesis of sandwich interspersed LaCO_3_OH/Co_3_O_4_/graphene oxide composite and the enhanced catalytic performance for methane combustion. Catal. Today.

[14] Sawicka-Chudy P., Sibiński M., Wisz G., Rybak-Wilusz E., Cholewa M. (2018). Numerical analysis and optimization of Cu_2_O/TiO_2_, CuO/TiO_2_, heterojunction solar cells using SCAPS. J. Phys. Conf. Ser..

[15] Wu R., Fan G.C., Jiang L.P., Zhu J.J. (2018). Peptide-based photoelectrochemical cytosensor using a hollow-TiO_2_/EG/ZnIn_2_S_4_ cosensitized structure for ultrasensitive detection of early apoptotic cells and drug evaluation. ACS Appl. Mater. Interfaces.

[16] Chen J., Kong L., Sun X.K., Feng J.H., Chen Z.W., Fan D.W., Wei Q. (2018). Ultrasensitive photoelectrochemical immunosensor of cardiac troponin I detection based on dual inhibition effect of Ag@Cu_2_O core-shell submicron-particles on CdS QDs sensitized TiO_2_ nanosheets. Biosens. Bioelectron..

[17] Pang X.H., Bian H.J., Wang W.J., Liu C., Khan M.S., Wang Q., Qi J.N., Wei Q., Du B. (2017). A bio-chemical application of N-GQDs and g-C_3_N_4_ QDs sensitized TiO_2_ nanopillars for the quantitative detection of pcDNA3-HBV. Biosens. Bioelectron..

[18] Yang L.W., Liu X.Q., Li L.L., Zhang S., Zheng H.J., Tang Y.F., Ju H.X. (2019). A visible light photoelectrochemical sandwich aptasensor for adenosine triphosphate based on MgIn_2_S_4_-TiO_2_ nanoarray heterojunction. Biosens. Bioelectron..

[19] Shu J., Qiu Z.L., Zhuang J.Y., Xu M.D., Tang D.P. (2015). In situ generation of electron donor to assist signal amplification on porphyrin-sensitized titanium dioxide nanostructures for ultrasensitive photoelectrochemical immunoassay. ACS Appl. Mater. Interfaces.

[20] Liu X.Q., Huo X.H., Liu P.P., Tang Y.F., Xu J., Liu X., Zhou Y.M. (2017). Assembly of MoS_2_ nanosheet-TiO_2_ nanorod heterostructure as sensor scaffold for photoelectrochemical biosensing. Electrochim. Acta.

[21] Da P.M., Li W.J., Lin X., Wang Y.C., Tang J., Zheng G.F. (2014). Surface plasmon resonance enhanced real-time photoelectrochemical protein sensing by gold nanoparticle-decorated TiO_2_ nanowires. Anal. Chem..

[22] Pang X.H., Bian H.J., Su M.H., Ren Y.Y., Qi J.N., Ma H.M., Wu D., Wei Q. (2017). Photoelectrochemical cytosensing of RAW264.7 macrophage cells based on a TiO_2_ nanoneedls@MoO_3_ array. Anal. Chem..

[23] Zhang R., Zhong X., Chen A.Y., Liu J.L., Li S.K., Chai Y.Q., Zhuo Y., Yuan R. (2019). Novel Ru (bpy)_2_ (cpaphen)^2+^/TPrA/TiO_2_ ternary ECL system: An efficient platform for the detection of glutathione with Mn^2+^ as substitute target. Anal. Chem..

[24] Zhou Y., Wang H.J., Zhuo Y., Chai Y.Q., Yuan R. (2017). Highly efficient electrochemiluminescent silver nanoclusters/titanium oxide nanomaterials as a signal probe for ferrocene-driven light switch bioanalysis. Anal. Chem..

[25] Tang Y.F., Liu X.Q., Zheng H.J., Yang L.W., Li L.L., Zhang S., Zhou Y.M., Alwarappan S. (2019). A photoelectrochemical aptasensor for aflatoxin B1 detection based on an energy transfer strategy between Ce-TiO_2_@MoSe_2_ and Au nanoparticles. Nanoscale.

[26] Liu N.N., Chen S.H., Li Y.L., Dai H., Lin Y.Y. (2017). Self-enhanced photocathodic matrix based on poly-dopamine sensitized TiO_2_ mesocrystals for mycotoxin detection assisted by a dual amplificatory nanotag. New J. Chem..

[27] Dai H., Xu G.F., Zhang S.P., Hong Z.S., Lin Y.Y. (2015). A ratiometric biosensor for metallothionein based on a dual heterogeneous electro-chemiluminescent response from a TiO_2_ mesocrystalline interface. Chem. Commun..

[28] Qiu Z.L., Tang D.P. (2020). Nanostructure-based photoelectrochemical sensing platforms for biomedical applications. J. Mater. Chem. B.

[29] So S., Hwang I., Schmuki P. (2015). Hierarchical DSSC structures based on “single walled” TiO_2_ nanotube arrays reach a back-side illumination solar light conversion efficiency of 8%. Energy Environ. Sci..

[30] Fan K., Chen J.N., Yang F., Peng T.Y. (2012). Self-organized film of ultra-fine TiO_2_ nanotubes and its application to dye-sensitized solar cells on a flexible Ti-foil substrate. J. Mater. Chem..

[31] Ma L.L., Yue Z., Huo G.N., Zhang S.S., Zhu B.L., Zhang S.M., Huang W.P. (2020). 3D Hydrogen Titanate Nanotubes on Ti Foil: A Carrier for Enzymatic Glucose Biosensor. Sensors.

[32] Shen S.H., Chen J., Wang M., Sheng X., Chen X.Y., Feng X.J., Mao S.S. (2018). Titanium dioxide nanostructures for photoelectrochemical applications. Prog. Mater. Sci..

[33] Chen Y., Li X., Cai G.N., Li M.J., Tang D.P. (2021). In situ formation of (0 0 1) TiO_2_/Ti_3_C_2_ heterojunctions for enhanced photoelectrochemical detection of dopamine. Electrochem. Commun..

[34] Cai G.N., Yu Z.Z., Ren R.R., Tang D.P. (2018). Exciton–plasmon interaction between AuNPs/graphene nanohybrids and CdS quantum dots/TiO_2_ for photoelectrochemical aptasensing of prostate-specific antigen. ACS Sens..

[35] Shu J., Qiu Z.L., Lv S.Z., Zhang K.Y., Tang D.P. (2018). Plasmonic enhancement coupling with defect-engineered TiO_2–x_: A mode for sensitive photoelectrochemical biosensing. Anal. Chem..

[36] Yang Z., Xu W., Yan B.D., Wu B.Q., Ma J.X., Wang X.H., Qiao B., Tu J.C., Pei H., Chen D.L. (2022). Gold and Platinum Nanoparticle-Functionalized TiO_2_ Nanotubes for Photoelectrochemical Glucose Sensing. ACS Omega.

[37] Cui L., Shen J.Z., Li C.C., Cui P.P., Luo X.L., Wang X.L., Zhang C.Y. (2021). Construction of a dye-sensitized and gold plasmon-enhanced cathodic photoelectrochemical biosensor for methyltransferase activity assay. Anal. Chem..

[38] Matković A., Petritz A., Schider G., Krammer M., Kratzer M., Karner-Petritz E., Fian A., Gold H., Gärtner M., Terfort A. (2020). Interfacial band engineering of MoS_2_/gold interfaces using pyrimidine-containing self-assembled monolayers: Toward contact-resistance-free bottom-contacts. Adv. Electron. Mater..

[39] Cui Z.K., Zhang L.X., Wang Y., He W.W. (2022). Plasmon excitation facilitating generation of electrons and reactive oxygen species for broad spectrum photocatalytic activity. Appl. Surf. Sci..

[40] Liu N., Mesch M., Weiss T., Hentschel M., Giessen H. (2010). Infrared perfect absorber and its application as plasmonic sensor. Nano Lett..

[41] Zaman M.A., Padhy P., Hesselink L. (2019). Solenoidal optical forces from a plasmonic Archimedean spiral. Phys. Rev. A.

[42] Wu L., Li F., Xu Y.Y., Zhang J.W., Zhang D.Q., Li G.S., Li H.X. (2015). Plasmon-induced photoelectrocatalytic activity of Au nanoparticles enhanced TiO_2_ nanotube arrays electrodes for environmental remediation. Appl. Catal. B Environ..

[43] Melvin A.A., Illath K., Das T., Raja T., Bhattacharyya S., Gopinath C.S. (2015). M–Au/TiO_2_ (M = Ag, Pd, and Pt) nanophotocatalyst for overall solar water splitting: Role of interfaces. Nanoscale.

[44] Ali H., Bensaali F., Jaber F. (2017). Novel approach to non-invasive blood glucose monitoring based on transmittance and refraction of visible laser light. IEEE Access.

[45] An H.Q., Zhu B.L., Li J.X., Zhou J., Wang S.R., Zhang S.M., Wu S.H., Huang W.P. (2008). Synthesis and characterization of thermally stable nanotubular TiO_2_ and its photocatalytic activity. J. Phys. Chem. C.

[46] Zhang L., Yu W., Han C., Guo J., Zhang Q.H., Xie H.Y., Shao Q., Sun Z.G., Guo Z.H. (2017). Large scaled synthesis of heterostructured electrospun TiO_2_/SnO_2_ nanofibers with an enhanced photocatalytic activity. J. Electrochem. Soc..

[47] Pustovalova A., Boytsova E., Aubakirova D., Bruns M., Tverdokhlebov S., Pichugin V. (2020). Formation and structural features of nitrogen-doped titanium dioxide thin films grown by reactive magnetron sputtering. Appl. Surf. Sci..

[48] Wang H.Q., Wu D.Y., Liu C.Y., Guan J.R., Li J.Z., Huo P.W., Liu X.L., Wang Q., Yan Y.S. (2018). Fabrication of Ag/In_2_O_3_/TiO_2_/HNTs hybrid-structured and plasma effect photocatalysts for enhanced charges transfer and photocatalytic activity. J. Ind. Eng. Chem..

[49] Zhou H., Zhang Y.R. (2014). Electrochemically self-doped TiO_2_ nanotube arrays for supercapacitors. J. Phys. Chem. C.

[50] Han Q.W., Zhang D.Y., Guo J.L., Zhu B.L., Huang W.P., Zhang S.M. (2019). Improved catalytic performance of Au/α-Fe_2_O_3_-like-worm catalyst for low temperature CO oxidation. Nanomaterials.

[51] Tahir M., Tahir B., Amin N.A.S. (2017). Synergistic effect in plasmonic Au/Ag alloy NPs co-coated TiO_2_ NWs toward visible-light enhanced CO_2_ photoreduction to fuels. Appl. Catal. B Environ..

[52] Ge L.Y., Hou R., Cao Y., Tu J.C., Wu Q. (2020). Photoelectrochemical enzymatic sensor for glucose based on Au@C/TiO_2_ nanorod arrays. RSC Adv..

[53] Yan B.D., Zhao X.R., Chen D.L., Cao Y., Lv C.Z., Tu J.C., Wang X.H., Wu Q. (2020). Enhanced photoelectrochemical biosensing performance for Au nanoparticle–polyaniline–TiO_2_ heterojunction composites. RSC Adv..

[54] Mai H.H., Janssens E. (2020). Au nanoparticle–decorated ZnO nanorods as fluorescent non-enzymatic glucose probe. Microchim. Acta.

[55] Long M., Tan L., Liu H.T., He Z., Tang A.D. (2014). Novel helical TiO_2_ nanotube arrays modified by Cu_2_O for enzyme-free glucose oxidation. Biosens. Bioelectron..

[56] Yang W.K., Xu W., Wang Y.D., Chen D.L., Wang X.H., Cao Y., Wu Q., Tu J.C., Zhen C. (2020). Photoelectrochemical glucose biosensor based on the heterogeneous facets of nanocrystalline TiO_2_/Au/glucose oxidase films. ACS Appl. Nano Mater..

[57] Artigues M., Abellà J., Colominas S. (2017). Analytical parameters of an amperometric glucose biosensor for fast analysis in food samples. Sensors.

